# Nutritional Status and Anthropometric Indices in High School Girls in Ilam, West Iran

**DOI:** 10.1155/2016/4275148

**Published:** 2016-06-15

**Authors:** Fatemeh Jamalikandazi, Elham Ranjbar, Eskandar Gholami-Parizad, Zeinab Ghazanfari, Seyed-Ali Mostafavi

**Affiliations:** ^1^Department of Public Health, Faculty of Health, Ilam University of Medical Sciences, Ilam, Iran; ^2^Department of Food and Drug, Faculty of Medicine, Ilam University of Medical Sciences, Ilam 7419654459, Iran; ^3^Prevention of Psychosocial Injuries Research Center, Ilam University of Medical Sciences, Ilam, Iran; ^4^Department of Health Education and Promotion, Faculty of Health, Ilam University of Medical Sciences, Ilam, Iran; ^5^Psychiatry Research Center, Roozbeh Hospital, Tehran University of Medical Sciences, Tehran, Iran; ^6^Department of Clinical Nutrition, School of Nutrition and Dietetics, Tehran University of Medical Sciences, Tehran, Iran

## Abstract

*Background*. Adolescence is one of the most challenging periods for human growth and nutritional status. The aim of this study was to assess the nutritional status and anthropometric indices in high school girls in Ilam.* Methods*. This cross-sectional study was performed on 360 domestic high school girl students chosen randomly by cluster sampling. Data were gathered through interviews performed by a dietitian to fill 24-hour dietary recall and food frequency and demographic questionnaires. Then we performed the anthropometric measurements and we compared the results with CDC2000 standards. We analyzed our data by N4 food analyzer and SPSS16 software.* Results*. The prevalence of obesity and overweight was 5% and 10.8%, respectively. Simultaneously, the prevalence of underweight was 20.2%. The prevalence of stunting was 5.8%. We also showed that 50% of high school girls in Ilam suffered from severe food insecurity, 14.7% suffered from mild insecurity, and 4.7% get extra energy from foods. Food analysis showed that micronutrients such as zinc, iron, calcium, folate, fiber, magnesium, and vitamin B12 were less than what is recommended by the RDA.* Conclusion*. Undernutrition and overnutrition are completely prevalent among girls studied in Ilam. This needs further acts and investigations in the field and more nutritional and health educations.

## 1. Introduction

Height and weight growth of each population are affected by factors that some of them are specific to that region. Some of these factors include heredity, race, economic status, and cultural and nutritional characteristics of that population [1]. Nutritional deficiencies are considered as the most important global problem. The easiest and most practical method for assessing nutritional problems in different groups of the population is using anthropometric measurements [2, 3].

Adolescence is distinguished from other life spans with features of rapid growth and increased need for nutrients [4, 5]. Additionally, 20% of height growth, 50% of weight growth, and 50% of adulthood skeletal mass are formed during this period [4, 6]. Researchers in Iran and other countries have indicated a poor nutritional status in this age and sex-specific group while growth spurts and especially menstruation in adolescent girls increase the nutritional needs dramatically [2].

One of the nutritional disorders increasing rapidly among adolescents is overweight or obesity, which is considered as background for adulthood obesity [7]. Prevalence of overweight among high school students in an Iranian national sample (CASPIAN study) increased to 11.2% in 2004. As a result, new cases of children with the metabolic syndrome may appear which in turn is likely to create an enormous economic and public health burden for Iran in the near future [8]. The National Food Consumption Survey conducted in 2001–2003 showed that the prevalence of thinness, preobesity, and obesity among girls aged 15 to 19 were 10.5%, 9.7%, and 3.9%, respectively [9].

Malnutrition in this period of life either as a form of overweight or underweight is the indicator of disease and predictor of morbidity and mortality in adulthood [10].

One simple, cheap, and relatively correct way of nutritional assessment is measuring anthropometric indices [11, 12]. The aim of this study was to determine nutritional status based on anthropometric indices and dietary intake of high school girls in Ilam, West Iran.

## 2. Materials and Methods

This cross-sectional study was performed on 360 high school students (14–18 years) chosen randomly by cluster sampling from 10 schools in the city of Ilam. At the beginning, 10 schools were randomly selected from 30 schools in Ilam. Then, we chose one class in each school randomly from each three educational grades. Then we randomly selected 12 students in each class and enrolled them in this study.

Before the study began, we received all necessary permissions from Ilam Province Education Office. Participants were recruited with informed written consent.

Students completed the demographic questionnaire which included information on age, level of education, family size, birth order, and mother's and father's education. We used a digital scale and a nonelastic tape to measure subject's weight (in kg) and height (in cm), respectively, in the standard situation. After measuring the height and weight, we calculated the body mass index (BMI). BMI was calculated as weight in kilogram divided by height in meter square. Underweight was defined as having a BMI lower than 5th percentile for age- and sex-specific BMI (CDC 2000-Center for Disease Control and Prevention); normal weight was defined as BMI between 5th and 85th percentile; at-risk for overweight and overweight were defined as BMI between 85th and 95th and greater than 95th percentile, respectively [13]. For the purpose of simplicity, CDC between 85th and 95th percentile is referred to as overweight and that greater than 95th percentile is referred to as obesity in this paper.

To collect data of dietary intake 24-hour food recall was performed by a dietitian. To decrease day-to-day variation of food intake this task was completed for 3 days. Food portions and sizes as large, medium, and small spoons, cups, and glasses were presented to students to help them correctly estimate their intake [14]. Because food recall relies on memory and to gather information on dietary habits, food frequency questionnaire (FFQ) was used to reduce the error rate. After collecting the food recall notes nutrient intake was analyzed by software N4. The macronutrients like carbohydrates, proteins, fats, and essential micronutrients such as vitamins B (B_9_ and vitamin B_12_), minerals (iron, calcium, magnesium, zinc, etc.), and the fiber were calculated [15]. We compared the nutrient intakes with Recommended Dietary Allowances (RDA). We then grouped nutrients intake as less than 75% of the recommended amounts and higher than 75% of the recommended amounts. According to this grouping, when at least 20% of population get less than 75% of recommendations, supplementation grow to be a priority in that society [16].

Data were gathered and we used SPSS ver. 16 software for statistical analysis.

## 3. Results

The results are presented in 3 tables. Data related to the percentage of participants in each educational grade, parents' education level, family size, and birth order is given in Table 1. Also, mean age, weight, height, and BMI of participants are separately listed in Table 2.

Findings of this study showed that prevalence of underweight in the total study population was 20.23% (5.1%, 10.6%, and 45% in the first, second, and third educational grade, resp.). The prevalence of overweight and obesity was 10.86% and 5%, respectively. The prevalence of stunting in this study was 5.8%.  Table 3 shows that highest prevalence of stunting was seen in third educational grade (10% less than 3rd percentile of height for 16–18 years of age).

In terms of energy, 50 percent of high school girls in the study suffered from severe food insecurity, 14.7 percent suffered from a slight insecurity, and 4.7 percent got extra energy. According to food analysis, dietary intake of micronutrients such as zinc, iron, calcium, folate, fiber, magnesium, and vitamin B_12_ was less than 75% of the RDA in the majority of subjects (Table 4).

## 4. Discussion

Wasting prevalence in the present study was 20.23%. This was similar to study performed by Gholami Parizad et al. [17] in Ilam and Mousavi et al. study [18] on 10–18-year-old girls in East Tehran and was dissimilar to Jaafari Rad et al. [19] conducted on 14–18-year-old girls in Sari and Esfar Jani et al. [20] study conducted on teenage girls in Tehran. Prevalence rates of wasting in these four studies were 23%, 30.7%, 3.8%, and 5.2%, respectively. Similarities in sociodemographic status and dietary habits may be responsible for nearly similar statistics.

In our study, the prevalence of obesity and overweight were 5% and 10.86%, respectively. Prevalence of obesity and overweight in earlier studies in Ilam [17], East Tehran [18], Sari [19], Tehran [20], Birjand [10], Tabriz [21], and Larijan [22], respectively, were 4% and 10%; 4% and 13%; 3.3% and 13.3%; 3.4% and 18.1%; 1.8% and 7.1%; 3.6% and 11.1%; and 5.3% and 14.8%. In a study conducted in Qatar [23], prevalence of obesity and overweight were 4.7% and 18.7%, respectively. In Turkey prevalence of obesity and overweight in 12–17-year-old girls were 2.1% and 10.6%, respectively [24]. Sadeqipoor and colleagues [25] in a study conducted in 1377 reported 13.7% of obesity rate in adolescent girls in Tehran (District 12). The prevalence of obesity in teenage girls of the Ilam city after Lahijan [22] was higher than any other city [8, 14–18, 20–22]. Prevalence of overweight in the city of Tabriz [21], Lahijan [22], and Sari [19] was more than teenage girls in Ilam. Differences in sociodemographic status people in these regions may be responsible for this. This prevalence was also higher in Qatar [23], Turkey [24], and United States of America [14] compared with teenage girls in Ilam.

The prevalence of stunting in our study was 5.8 percent. This rate was 23% in 1388 in Ilam [17]. The prevalence of stunting among girls has been reduced dramatically in Ilam compared to 1388. This may indicate an increased awareness of families and improved nutritional status of pupils in Ilam. Jaafari Rad et al. reported 13.3% of stunting among teenage girls in Sari [19] which is more than twice higher than the prevalence of stunting in Ilam. On the other hand prevalence of wasting in Ilam was the second after Tehran. Possible reasons for remarkable differences in the results of the above researches may be geographical, economic, and cultural differences and different methods in measurements and classifications.

In regard to energy intake, if a population gets 81–90% of the caloric energy needed it may experience slight insecurity. Calories access less than 80% of what is needed is the indicator of severe insecurity. And more than 120% of caloric need access is considered as overload [26]. We grouped nutrients intake as less than 75% of the recommended amounts and higher than 75% of the recommended amounts. According to this grouping, when at least 20% of population get less than 75% of recommendations, supplementation grows to be a priority in that society [16]. In our study about 50 percent of adolescents were in the extreme insecurity of caloric intake (less than 80% of energy intake compared to RDA). About 14.7 of them experienced slight insecurity and 4.7 percent got extra energy. Comparing our results with the survey that was conducted on adolescents in Sari [27] is showing a more unfavorable situation. Approximately 11.7% of teenage girls had energy intakes less than 80% of RDA. In a study conducted on adolescents in rural India about 70 percent of children get less than 70 percent of the RDA for energy [25]. In another study, only 9% of Bangladeshi adolescents were in accordance with the recommended energy intake [28]. In a study of adolescents conducted in Districts 3 and 6 of Tehran, 36% and 42.5% (resp.) had lower energy intakes of 90th percentile of RDA, while 25.3% and 21.9% received extra energy [20]. Urban and rural adolescents of Bardsir 47.7% and 50%, respectively, were deficient in energy intake [26]. Thus, we found that lack of energy (less than 90% RDA) in adolescent girls in Ilam was 64.7%, which is near the same energy shortage in India [25] and rural adolescents in Bardsir [26].

Table 4 shows that many of students in Ilam have deficiencies in vitamins B_9_ and B_12_ and minerals such as magnesium, calcium, zinc, iron, and fiber. In a study of the adolescents in Bangladesh, deficiency of vitamins and minerals such as iron, calcium, vitamin A, vitamin C, and Riboflavin was observed [25]. In Hindi [29], adolescents low intakes of iron, calcium, vitamin A, vitamin C, and riboflavin have been shown. In Asian American adolescent girls low calcium intake has been reported [16]. Tehran District 16 adolescents also received low levels of calcium, phosphorus, and vitamin B_2_ [28]. Adolescents in Sari also have deficiencies in vitamins and minerals such as vitamin A, vitamin B_2_, vitamin C, folacin, B_12_, calcium, and phosphorus [27]. Thus, it is clear that the lack of nutrients intake can be seen in the majority of adolescent girls in all regions. Differences in nutrient intakes in regions may be due to different dietary patterns that are common in that area and socioeconomic differences. However, calcium deficiency is very common among all adolescents in all regions [27]. This may be due to increased needs for calcium in teenage girls and low intake of calcium sources especially milk. Gossips and fears of contamination of milk may contribute to this issue.

In general, it seems that micronutrient insufficiency intake is one of the major problems in teenage girl students in all parts of the world and also has been seen in Ilam.

## 5. Conclusion

Undernutrition or underweight and on the other hand overnutrition or overweight are completely prevalent among girls studied and evidenced by our results. These extremes need further investigations in the field and more nutritional and health education and field trials.

## Suggestions

Adolescence is one of the life's most important periods for mental, emotional, and physical growth. So it is necessary to achieve a healthy diet and intake of macronutrients and micronutrients and the anthropometric indices always should be monitored. To meet this goal, localization of height, weight, and BMI curves is highly suggested by using information of studies performed in this area.

## Figures and Tables

**Table 1:** Demographic characteristics of participants.

Variable	*N* (%)
Educational grade	
Grade 1	118 (32.7)
Grade 2	122 (33.8)
Grade 3	120 (33.3)
Parents education	
Father	
Illiteracy	65 (18.1)
Low education	155 (43)
Diploma	88 (24.4)
Associate degree	23 (6.4)
Bachelor's degree or higher degree	29 (8.1)
Mother	
Illiteracy	77 (21.4)
Low education	198 (54.9)
Diploma	65 (18.1)
Associate degree	11 (3.1)
Bachelor's degree or higher degree	9 (2.5)
Birth order	
1st	103 (28.6)
2nd	72 (20)
3rd	52 (14)
4th	50 (13.9)
5th	26 (7.2)
6th+	57 (15.8)
Family size	
3-4	52 (15.5)
5-6	183 (50.8)
+7	125 (34.8)

**Table 2:** Mean age, weight, height, and BMI of high school girls in Ilam.

	Age (mean ± SD)	Weight (mean ± SD)	Height (mean ± SD)	BMI (mean ± SD)
Grade 1	14 ± 0.9	54 ± 12.3	160 ± 7	20.9 ± 3.9
Grade 2	15.7 ± 0.5	55.4 ± 10.9	161 ± 5.6	21.1 ± 3.7
Grade 3	16.7 ± 0.6	56 ± 11.1	162 ± 6.3	21.5 ± 3.9

Total	15.76 ± 1.08	55.1 ± 11.4	161 ± 6.2	21.1 ± 3.9

**Table 3:** Distribution of stunting, wasting, obesity, and overweight in different educational levels.

	BMI percentile				Assessing height		
	<5 (wasting)	5–<85 (normal weight)	85–<95 (overweight)	>95 (obesity)	3> (stunting)	3–97 (normal)	>97 (overheight)
Grade 1	6 (5.1)	89 (75.4)	14 (11.9)	9 (7.6)	6 (5.1)	109 (92.4)	3 (2.5)
Grade 2	13 (10.6)	91 (74.6)	13 (10.7)	5 (4.1)	3 (2.5)	119 (97.5)	—
Grade 3	54 (45)	50 (41.7)	12 (10)	4 (3.3)	12 (10)	108 (90)	—

**Table 4:** Comparison of micronutrient intakes of high school girls in Ilam with RDA.

	Less than 75% of RDA *N* (%)	More than 75% of RDA *N* (%)
Vitamin B_12_	239 (66.4)	121 (33.6)
Vitamin B_9_	321 (89.2)	39 (10.8)
Vitamin B_1_	15 (5)	342 (95)
Vitamin B_2_	80 (22.2)	280 (77.8)
Vitamin B_3_	50 (13.9)	310 (86.1)
Vitamin B_6_	151 (41.9)	209 (58.1)
Vitamin A	179 (49.7)	181 (50.3)
Vitamin C	98 (27.2)	262 (72.8)
Zinc	212 (58.9)	148 (41.1)
Fe	122 (33.9)	238 (66.1)
Ca	337 (93.6)	23 (6.4)
Mg	257 (71.4)	103 (28.6)

## Additional Points

Regarding the fact that target study population was school students, sampling and data collection should be done in certain situations and when they do not interfere with the education programs in schools.
